# Human-machine interactions with clinical phrase prediction system, aligning with Zipf’s least effort principle?

**DOI:** 10.1371/journal.pone.0316177

**Published:** 2024-12-31

**Authors:** Jamil Zaghir, Mina Bjelogrlic, Jean-Philippe Goldman, Julien Ehrsam, Christophe Gaudet-Blavignac, Christian Lovis

**Affiliations:** 1 Division of Medical Information Sciences, Geneva University Hospitals, Geneva, Switzerland; 2 Department of Radiology and Medical Informatics, University of Geneva, Geneva, Switzerland; University of Kurdistan Hewler, IRAQ

## Abstract

The essence of language and its evolutionary determinants have long been research subjects with multifaceted explorations. This work reports on a large-scale observational study focused on the language use of clinicians interacting with a phrase prediction system in a clinical setting. By adopting principles of adaptation to evolutionary selection pressure, we attempt to identify the major determinants of language emergence specific to this context. The observed adaptation of clinicians’ language behaviour with technology have been confronted to properties shaping language use, and more specifically on two driving forces: conciseness and distinctiveness. Our results suggest that users tailor their interactions to meet these specific forces to minimise the effort required to achieve their objective. At the same time, the study shows that the optimisation is mainly driven by the distinctive nature of interactions, favouring communication accuracy over ease. These results, published for the first time on a large-scale observational study to our knowledge, offer novel fundamental qualitative and quantitative insights into the mechanisms underlying linguistic behaviour among clinicians and its potential implications for language adaptation in human-machine interactions.

## Introduction

Human language is a complex and ever-evolving phenomenon, characterized by flexible form-meaning mappings in words. The theory of arbitrariness in language suggests that there is no inherent basis for these mappings [1–5]. This theory would allow for a wide range of word forms to be used, and examining these choices can provide insights into human’s cognitive processeshttps://www.zotero.org/google-docs/?MFjTGT [6].

In Shannon’s well-known communication framework [7], language can be seen as a system where spoken or written words act as codes that a listener or receiver deciphers to understand the intended message or meaning. Zipf, through the principle of least efforthttps://www.zotero.org/google-docs/?8OcWuT [8], suggests that language users tend to structure these codes in a way that maximises the efficiency required to convey information effectively. It implies that humans are inclined to minimise their cognitive efforts to achieve mutual understanding during communication. Thus, the most frequent words would be the shortest, and the rarest words would usually refer to sophisticated and intricate concepts [9]. This relationship between word frequency and length constitutes the main concept of Zipf’s Law of Abbreviation [10]. Moreover, there is a theoretical link between Zipf’s Law of Abbreviation and Zipf’s Law. Several studies have derived both laws from the principle of word length minimization combined with additional constraints, suggesting that these laws are interconnected through the concept of compression in language [9, 11]. Zipf’s Law states that in each corpus of natural language, the frequency of any word is inversely proportional to its rank in the frequency table, meaning that a few words are used very often while many others are used rarely.

Zipf’s Law of Abbreviation manifests consistently across about a thousand languages encompassing eighty distinct linguistic families, underscoring the correlation between the length of a word and its frequency in written text [12]. Moreover, various studies support that these principles hold universally across languages [12, 13], and throughout the human lifespan [14]. Yet, this correlation seems to be relatively moderate. For example, numerous cases in English exist where short, infrequently used words, such as *ewe* and *pyx*, and longer, commonly used words, like *however* and *actually*, contradict this pattern. While the economy of expression seems to influence the lexicon, it is clear that word length minimization is not the sole factor: language users are engaged in many other pressures [15]. These pressures may be related to constraints inherent in the coding schemes of information theory. Building upon these insights, research by Kanwal et al. [16] investigates Zipf’s least effort principle and unveils through a synthetic three-word lexicon that humans exhibit a tendency to adapt their form-meaning mappings under the influence of two competing pressures: communication efficiency and accuracy. Communication efficiency focuses on conveying information with minimal effort, while communication accuracy aims to achieve successful and accurate communication [13, 17–20]. While the efficiency pressure explains humans’ inclination towards utilising shorter words more frequently, word length is not exclusively determined by frequency. Despite recent works challenging the role of information content, showing that frequency has a more robust correlation with length [21–23], a previous work [24] claims that the average predictability of words from context emerges as a more reliable predictor of length than mere frequency.

The predictability of a word closely intertwines with the information content it carries, and this, in turn, hinges upon the context it resides in. To illustrate this statement, let us consider the contrasting scenarios presented by the sentences involving the word *worm* [25]. In the sentence *"The early bird catches the worm"*, the word *worm* holds a lower surprise value due to its expected presence, resulting in reduced information content. However, in the sentence *"Our early bird special today is a baked-apple worm"*, the mention of *worm* becomes highly surprising within this context, elevating its information content. The higher the information content, the more the word needs to be mentioned to be understood by the receiver. The relationship between predictability, context, and information content holds particular significance, particularly in technical multi-word expressions commonly found in specialised domains such as medicine. As an example, the pathology *"Chronic obstructive pulmonary disease"* (commonly known as COPD) ranks as the third leading cause of death worldwide [26–29], impacting lung function and respiratory health significantly. This health issue being prevalent in hospitals globally, certain constituent words like *pulmonary* make a reduced contribution to the phrase’s informational significance in the medical context. In the case of the term *disease*, the information content approaches nearly zero.

In line with Zipf’s least effort principle favouring an optimal balance between efficient information transmission and accuracy, the ideal scenario revolves around employing short words brimming with high information content. A previous study shows a potential linear correlation between word length and quantity of information content [30]. Shorter words are more likely to have lower information content, and longer words might be associated with higher information content. Expanding beyond unigrams, Zipf’s law seems to apply to n-gram phrases [31, 32]. This hypothesis appears to be valid for the world’s two most widely spoken languages, English and Mandarin [31]. An earlier work goes further, asserting that the length rank distribution of phrases is closer to Zipf’s law than that of words [32].

As our linguistic landscape becomes increasingly influenced by technological advancements, such as OpenAI’s ChatGPT, the scope broadens to encompass the evolving nature of language use in the digital realm. Technology adoption, a cornerstone of our digital society, necessitates not only user acceptance [33–35] but also adaptation [36–38]. Research on user adaptation focuses on the behavioural efforts users make to cope with technological situations in their work environment, including their use of language. Therefore, investigating user adaptation to technology is critical for institutions willing to implement technologies. This adaptation extends to language itself, where users strategically modify their linguistic interactions to enhance their engagement with novel technological interfaces. This is exemplified by the fact that the use of GPT-based technologies can be improved through prompt engineering techniques [39, 40]. For instance, adding expressions like ’*Think step by step*’ at the end of the input to GPT-3 enabled it to engage in multi-step reasoning [41]. Additionally, this prompting method has emerged as a strategy to bolster language models’ performance in tackling complex tasks by deconstructing them into smaller, more manageable steps [42]. Instead of expecting an immediate comprehensive response, this prompt technique involves furnishing the model with examples to guide its reasoning process. Consequently, the model generates responses incrementally, using the provided examples as a guide when solving intricate problems. This approach has proven effective in enhancing ChatGPT’s capabilities in domains such as complex arithmetic, logical reasoning, and understanding context-based questions, which are generally resistant to traditional scaling improvements. The evolution of human language has transitioned into a distinct phase marked by the emergence of conversations involving artificial intelligences. The current landscape moves beyond the Turing test to interactions between intelligences, necessitating a crucial exploration of how these engagements shape the ongoing evolution of language. In this pursuit, language adaptation emerges as a critical facet, unravelling the strategies employed by users to enhance their interaction with a spectrum of technologies. This includes more elementary technologies such as spoken dialogue systems and autocomplete tools [43–46]. However, amidst this technological evolution, little is known about how language use evolves in these contexts and whether it aligns with the principles laid out in Zipf’s theories.

An intriguing direction to learn more about this question is the analysis of expression search technology activities within hospital settings, where physicians are mandated to engage with a phrase prediction tool for reporting patient admissions. Notably, these physicians traverse unfamiliar terrain as they lack in-depth knowledge of the inner workings of this technology. We therefore hypothesise that their interactions are undergoing a process of linguistic adaptation. This phenomenon could be accentuated in the clinical context, where swift interactions are imperative, and errors can bear critical and vital consequences. Our study gives attention to the chronological observation of how pressures related to efficiency and accuracy vary throughout this adaptation process. This empirical exploration serves as a platform to test hypotheses rooted in Zipf’s least effort principle. Specifically, for a given target label (e.g. pathology, health problem), we aim to designate an optimum set of queries, referred to as the Pareto front, which navigates the trade-off between conciseness and distinctiveness of the interactions. The study aims to discern the proximity or divergence of users’ language interactions from this optimal set. Additionally, our investigation extends to examining these two objectives, conciseness and distinctiveness, as two independent variables. This facet allows us to ascertain whether users tend to favour one objective over the other in their linguistic adaptations when engaging with the technology. By dissecting the two components, we aim to understand the extent to which each pressure contributes to users’ linguistic adaptation to the technological system that expects a free-text input.

## Materials and methods

### Technology involved in the study

**Fig 1:**
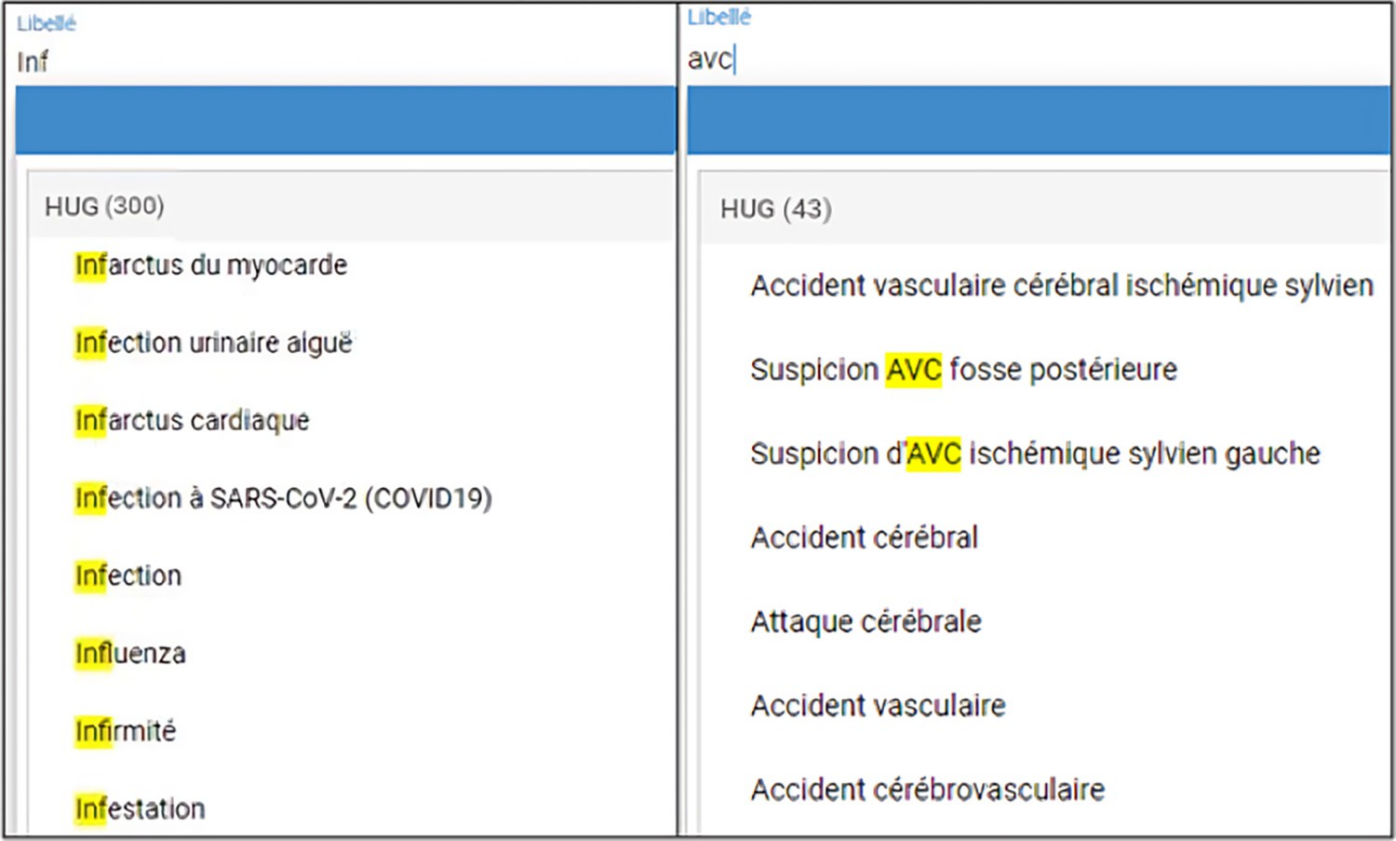
Automatic completion system in the HUG (two use cases).

The study is based on a phrase prediction tool used in the University Hospitals of Geneva (HUG) to investigate the pressures driving the evolution of language use in technological settings. In the hospitals, health data includes both free-text data, such as doctors’ notes, and categorical data, like lab results, and diagnosis codes. The use of such codes facilitates the secondary use of data where health professionals can repurpose the data collected for clinical care to support other activities like research, quality improvement, and public health monitoring [47–49]. To do so, the clinicians from the hospitals use internally-built terminologies such as patients’ health problems [50]. This problem list is designed to meet specific needs, including billing, logistics, and operative planning. More importantly, it bridges the gap between economic, logistic, financial, scientific, and health actors. It currently contains 25,349 labels in French, each associated with one or multiple synonyms and codes from different standards. As these professional users are under constant pressure, they work using a phrase prediction-based system (Fig 1) instead of scrolling down a constrained closed list, improving efficiency during problem selection through typed partial input. As illustrated in Fig 1, the phrase prediction tool suggests a set of candidate labels by leveraging text similarity, as well as considering synonyms of the labels in response to a query (in this case, *avc* is implemented as a synonym of the label *accident vasculaire cérébral*). The system is designed to be incremental. This means it dynamically updates and refines the list of label options as the user types. As the user enters more characters, the system progressively narrows down the list of possible labels, reducing the number of candidates based on the input provided. It is worth noting that the tool does not use any shortcut keys typically found in some modern implementations of writing systems, like those used in East Asian languages, which contributes to the perception that lower-ranked predictions should take more time and effort to select.

### Data description

Each time a hospital employee uses the tool, the name of the involved user, the partial input typed (the query), the selected label, the associated code of that label, and the date of that event are stored. Table 1 shows synthetic examples that could occur in the large real-world dataset. As part of this research work, the dataset is enriched. For each row, the three following features have been added:

- The position of the selected label in the list of suggestions at the selection time (Rank);
- The number of characters in the query (QLen);
- The user-label seniority. This refers to the number of times a specific user has chosen a particular label. It starts at 1 for the first interaction and increases by 1 each time the same user selects the same label again. Therefore, if a user-label seniority is k, it means the user has chosen that label k times.

**Table 1:** Synthetic examples from HUG phrase prediction dataset recording activities. The table describes the username, the query, the selected label, the ID of the label, the date, the rank, the length of the query, the seniority. This table being a toy example, the seniority values are intentionally initialized to 1 to illustrate the concept (meaning both J. Doe and B. Lee are selecting these labels for the first time).

User	Query	Selected Label	ID Label	Date	Rank	QLen	Seniority
J. Doe	diabet	Diabète de Type 2	4578	03/14/2022	1	6	7
J. Doe	diab	Diabète de Type 2	4578	03/15/2022	4	4	8
J. Doe	ac vas	Accident vasculaire cérébral ischémique	2477	03/15/2022	1	7	49
B. Lee	diab	Diabète de Type 2	4578	03/15/2022	4	4	87
J. Doe	diab	Diabète de Type 2	4578	03/17/2022	4	4	9
B. Lee	drs	Douleur rétrosternale	10598	03/18/2022	1	3	23
J. Doe	ivrs	Infection des voies respiratoires supérieures	847	03/20/2022	1	4	14

These features are represented in the three last columns of Table 1. This dataset contains about one year of phrase prediction activities with 183,098 entries, 1,763 involved users, and 10,774 different labels out of 25,349 from the HUG’s internal terminology.

### Multi objective optimisation (MOO)

The MOO domain encompasses a wide range of approaches [51, 52], and it is commonly observed that achieving optimal results for all objectives simultaneously is not feasible. Improving one objective may lead to a degradation of another. A review reveals a multitude of techniques for addressing MOO problems [52], however, two stand out for their balance of efficacy and computational efficiency: the Pareto method [53] and scalarization-based methods [54]. The former aims to find a set of dominant solutions (named the Pareto front) among the solutions. In the case of scalarization, it consists of converting the MOO problem into a single-objective optimisation problem by using weighted coefficients on objective functions. Our work presents an evaluation of the queries utilised by clinicians based on two properties through the resolution of a MOO problem: the conciseness and the distinctiveness of the queries.

### Solution domain of the MOO

We define ***Φ*** as the alphabet consisting of 38 symbols, including {*a* − *z*}, digits {0 − 9}, and the space character. We also define the set T, called **terminology**, as containing written expressions. Among elements from that set called **labels**, *L* is the length of the longest label.

The set Φ* represents all possible strings formed from ***Φ*** up to the maximum length *L*, excluding the empty string. Formally:

$$\Phi^{*}=\cup_{i=1}^{L}\Phi^{i}$$ (1)

Where ***Φ***^***i***^ denotes the set of strings of length *L*.

The size of the set Φ* is:

$$|\Phi^{*}|=\sum_{i=1}^{L}38^{i}=\frac{1-38^{L+1}}{1-38}-1$$ (2)

We define the set $A_{T}$ as containing all possible sequences without repetition of labels from T, ensuring no label is suggested twice by the prediction system. ${A_{T}}^{J}$ is the set of sequences from $A_{T}$ of size ***J***.

The **phrase prediction function** *F* maps elements *x*∈Φ* to $A_{T}$, where *F*(*x*)_*j*_ denotes the ***j***^***th***^ element outputted by *F*(*x*).

The solution space Φ* is narrowed down to ℙ*, containing elements empirically observed at least once in the phrase prediction database. ℙ^*k*^ is a subset of ℙ*, whose permutations are of length k.

The optimization problem aims to minimize two objective functions:

$$O_{E}(t)=\left\{ \begin{array}{l} argmin_{p^{k}\in P^{k}}[min_{k\in N^{*}}(F(p^{k}))]\;such\;that\;\exists j\in N^{*},j\leq J,F{\left(p^{k}\right)}_{j}=t\\ argmin_{p\in P^{*}}[min_{j\in N^{*},j\leq J}(F{\left(p\right)}_{j})]\;such\;that\;F{\left(p\right)}_{j}=t \end{array}\right.$$ (3)

The optimum is based on ℙ* instead of Φ* due to the high computational complexity required to compute the solution with Φ* (Eq 2). Furthermore, the optimal solution within Φ* often relied on the first digits of the label ID, information that is neither known, nor accessible to the users, whereas ℙ* encompasses queries that are accessible to users. Since this optimum is empirical, a caveat needs to be made: this optimum does not represent the absolute optimal solution, but users approaching it represents a form of improvement. The detailed version of the mathematical formulation of the problem is depicted in Section 4 of S1 File.

### MOO analysis

In the context of the function described in Eq 3, we consider two key components: the completion label *t* and the query *p*^*k*^. The completion label *t* represents a specific target outcome within the predicted completions (e.g., "*Back pain*"). Meanwhile, *p*^*k*^ denotes the shortest query from a set of queries ℙ*. For example, *p*^*k*^ could be "*back p*" (*p*^6^∈ℙ^6^), where ℙ^6^ represents all 6-character queries. The optimization function (Eq 3) shows two main objectives, for a given completion label *t*: the first function aims to identify the shortest query *p* such that the label *t* is among the corresponding predicted completions in F(*p*), while the second one looks for the query *p* such that the completion label *t* is ranked the highest among all corresponding predicted labels in F(*p*).

**Fig 2:**
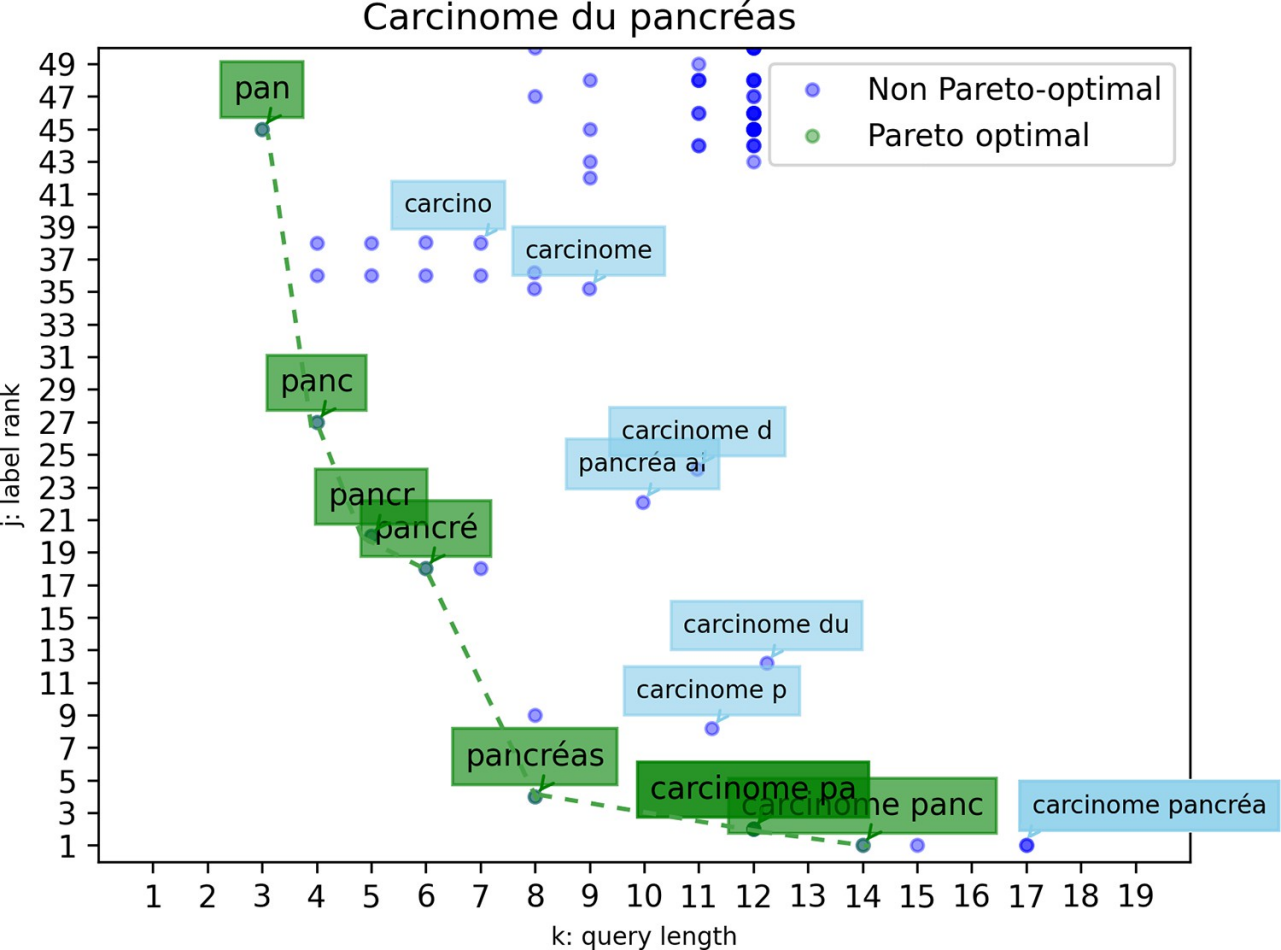
Pareto front computed for the label: *Carcinome du pancréas*. The dashed line represents the Pareto front, which simply connects all solutions represented by the green dots. The colour intensity of the dots indicates the frequency of the occurrences.

As the relative importance of the two objectives is not known, we use the Pareto method to solve the MOO problem. For each label from the terminology, the queries are plotted with respect to the objectives, and the Pareto front, the optimum, is computed according to Eq (3). Pareto identifies solutions in the two-objective optimization problem where no objective can be improved without worsening the other. As shown in Fig 2, the Pareto front is often represented by multiple points as there might be multiple solutions. Sometimes, as depicted in Section 2 of S1 File, the Pareto front is represented as one unique point in case there is one optimal solution (e.g. to select “*Accident de la voie publique*”, the query “*avp*” is the most optimal solution as it is short, and the label tops the ranking).

The Pareto front embodies optimal solutions, thus the Euclidean distance between a query and its nearest Pareto-optimal counterpart serves as a metric for gauging proximity to the optimum. A decrease in this Euclidean distance over time implies that user inputs get closer to the optimal set of queries. As each label possesses its own Pareto front, the chosen time axis is the user-label seniority.

Section 1.3 in S1 File demonstrates that the selected labels also exhibit a power-law distribution, implying that many labels are rarely chosen and have a low probability of attaining high seniority. To eliminate this bias due to the label variation across seniority levels, we exclude the labels that are absent in the highest seniority and examine the user behaviours with consistent labels over time. As we are working with a smaller sample of user-label pairs, it is essential to verify that the sample analysis is still representative of the whole population size. To ensure a representative sample of the population with a margin of error below 3% and a 95% confidence level, we chose a maximum user-label seniority level of 90 (detailed in Section 3 of S1 File).

## Results

### Progressive refinement of user interactions towards the optimum

For each available label in the problem list, we compute the Pareto front, a set of queries representing the best possible trade-off between query length and label rank simultaneously. This collection of queries represents the shortest possible length while maintaining the highest achievable rank for a given label. We introduce the concept of user-label seniority, reflecting users’ experience levels in selecting corresponding labels. User-label seniority serves as the chronological framework to analyse variations in efficiency and accuracy across users engaging the technology at different frequencies.

**Fig 3:**
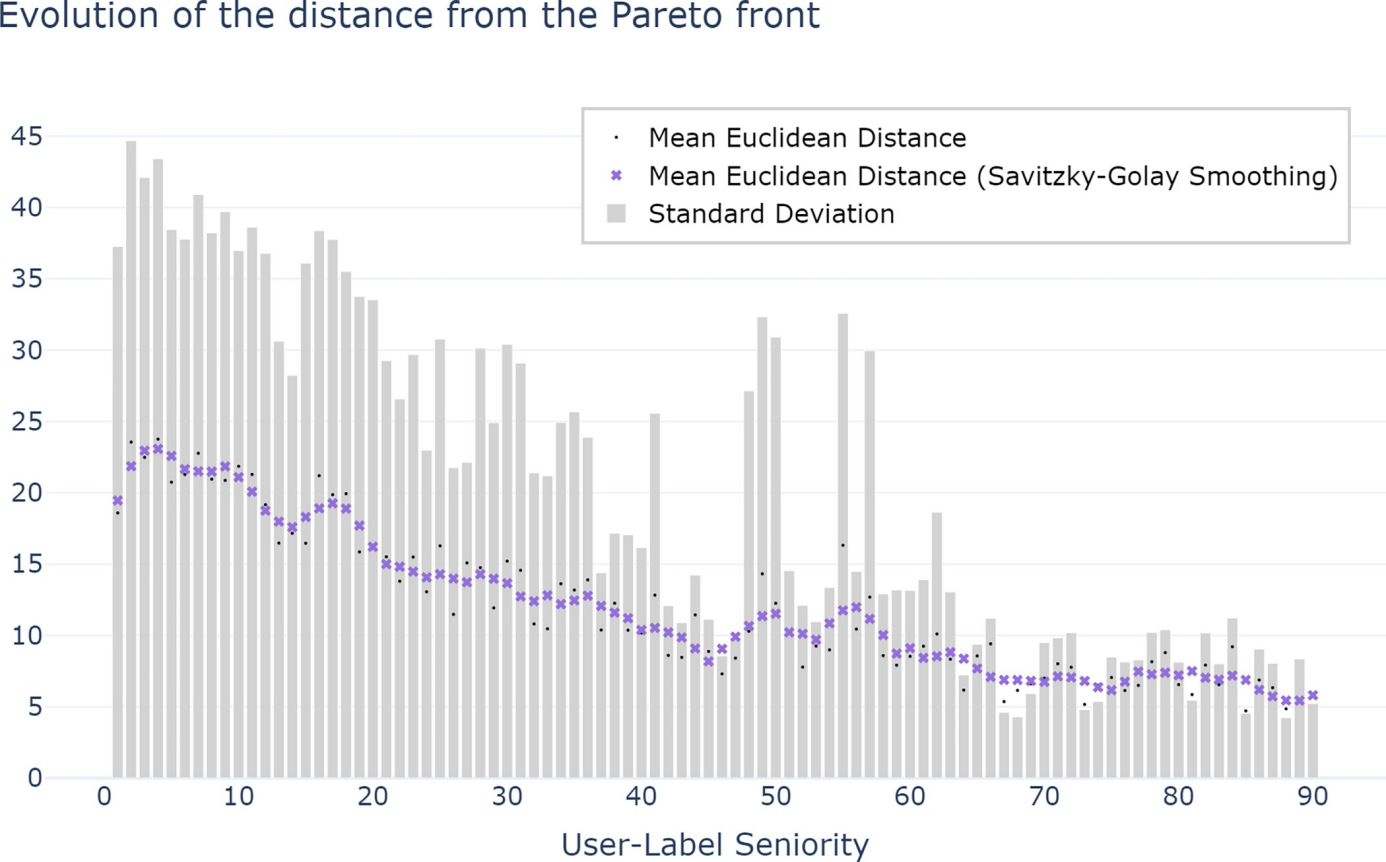
Distance of the queries from the Pareto front with respect to the user-label seniority. Each point includes all occurrences with the same user-label seniority.

Fig 3 illustrates the mean and standard deviation Euclidean distance (with and without Savitzky-Golay Smoothing) between users’ queries and the closest element from the Pareto front, where the Pareto front denotes the combination that minimises query length while achieving the best possible rank. This result reveals a discernible trend: as user-label seniority increases, users tend to approach the optimum, evidenced by a decrease in the average Euclidean distance from the Pareto front.

Additionally, the graph showcases the standard deviation, capturing the overall variability of distances between queries and the optimum at each user-label seniority level. Notably, as user-label seniority progresses, the standard deviation of the Euclidean distance from the Pareto front decreases significantly. At user-label seniority level 90, the standard deviation drops to a quarter compared to that calculated at the first user-label seniority level. This decrease clearly indicates a reduction in global variability across interactions as users gain experience. Our findings suggest a progressive refinement in users’ approaches, with higher user-label seniority correlating with greater proximity to the optimal query-label trade-off, demonstrating a convergence towards more efficient and accurate interactions.

### Adaptation driven by communication accuracy rather than communication efficiency

In our investigation, the Pareto front is used to optimise queries concerning two objectives: query length (conciseness) and label rank (distinctiveness). However, a critical question remains unanswered: what drives the convergence toward the Pareto front? Specifically, we aim to discern whether users prioritise shorter query lengths, achieving higher label ranks, or both, as they move towards this optimal front. To elucidate this, we designed an experiment to dissect and analyse these two objectives separately, seeking to uncover the primary force steering convergence toward the Pareto front.

**Fig 4:**
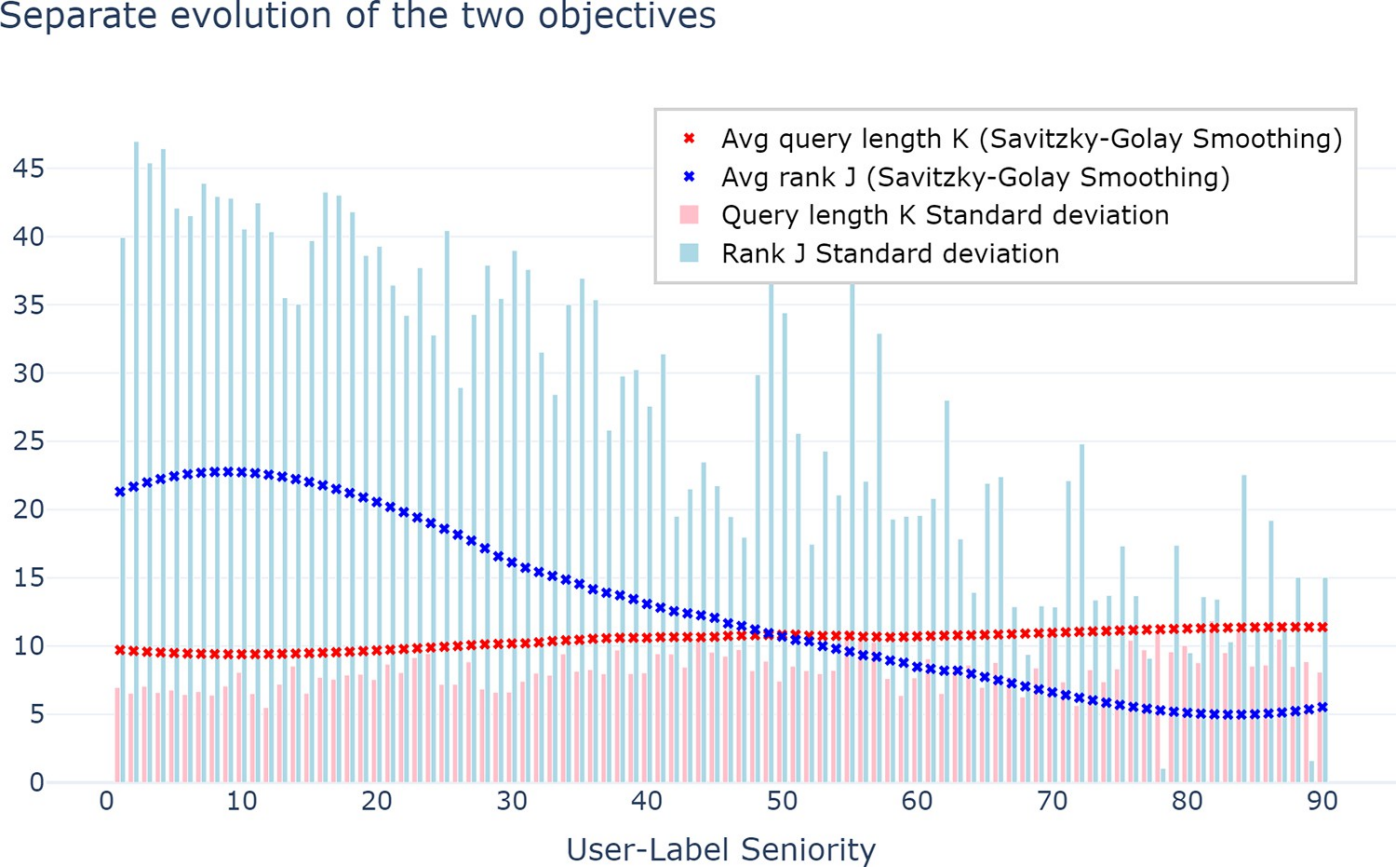
Monitoring the evolution of both objectives (Query length, label rank) separately.

Fig 4 presents a compelling insight: users’ query length does not diminish significantly, maintaining an average length between 10 and 12 characters. Contrastingly, the average rank of the chosen label experiences a notable decline as users gain familiarity and expertise with the label, plummeting from roughly 20th rank during initial selections to 5th rank on average by the 90th selection. This observation underscores a key trend: users tend to develop more refined and discriminating queries without substantially increasing query length, indicating a priority on communication accuracy over efficiency. As users become more adept, they demonstrate a marked ability to craft more precise queries without significant alterations in query length, signifying a prioritisation of communication accuracy in this phrase prediction task.

Examining standard deviations, the variability in query length remains relatively stable across different levels of user experience. Conversely, similarly to the convergence towards the Pareto front shown by Fig 3, the standard deviation associated with label rank exhibits a decreasing trend, indicating reduced variability as user experience advances.

### Evolution of the use of the medical idioms

Expressions and words that are part of the medical jargon are also employed by healthcare users in conjunction with the French language. While these expressions might not be easily comprehensible by laypeople, including patients [55], they often offer concise and semantically clear communication for healthcare professionals (e.g. “*BPCO*” means “*Bronchopneumopathie Chronique Obstructive*”, the French equivalent of COPD). We aim to look into the question of whether this adaptation of interactions could be significant enough to impact the use of medical jargon already familiar to users. To explore this, we carried out an experiment tracking the use of these expressions across users’ level of experience, to ascertain whether advanced users predominantly relied on queries from the medical jargon compared to beginners.

**Fig 5:**
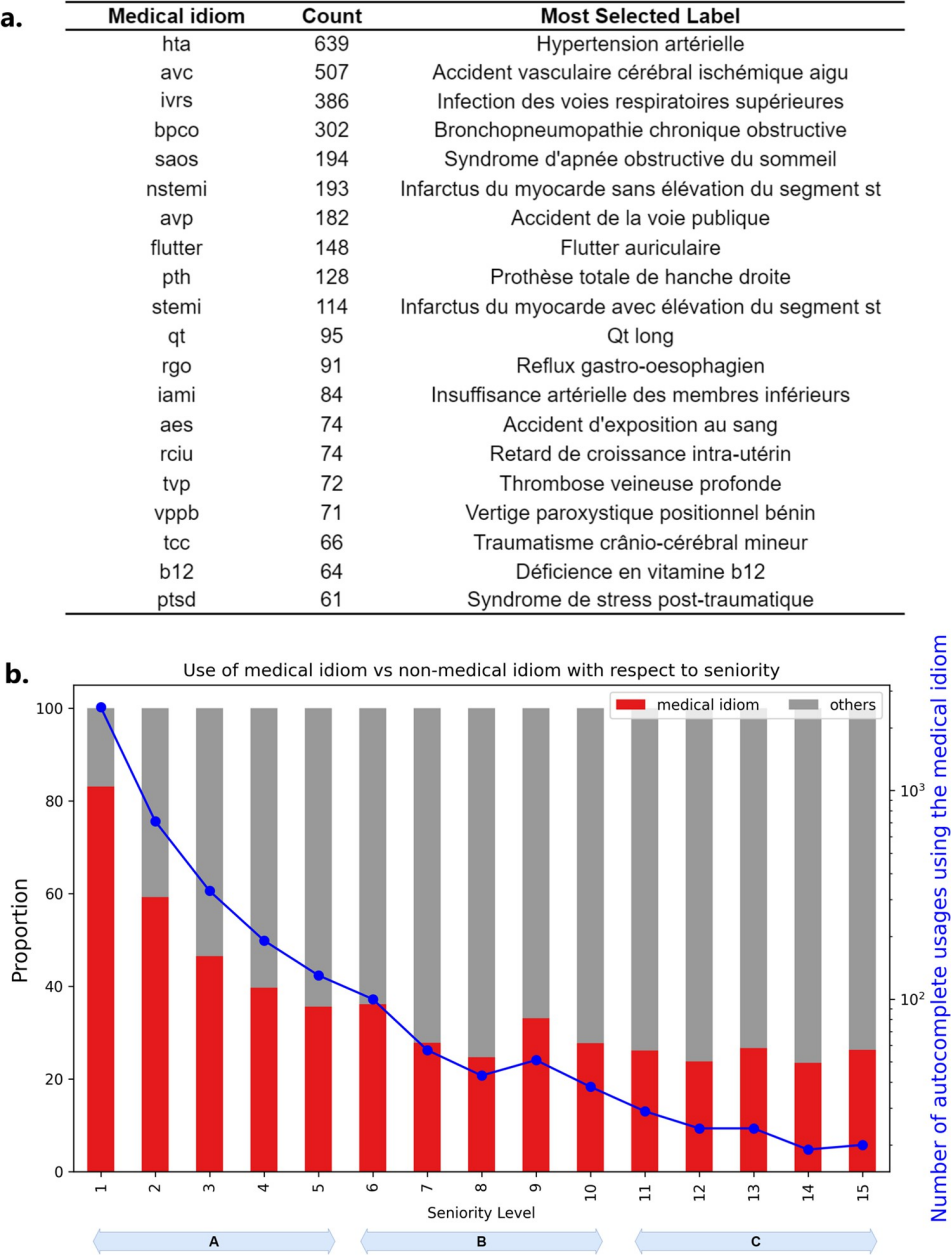
Use of acronyms, abbreviations and non-French words issued from medical idiom. a: Top 30 most used expressions from the medical idiom, with the number of occurrences and the most selected label for each expression. b: Proportion of queries from medical idiom against others by seniority level, for labels associated with at least one query from the medical idiom. Blue curve and the left y-axis indicate the amount of user activities using one of the 204 extracted expressions from the medical idiom.

We extracted a total of 204 expressions among the queries, mainly comprising acronyms and abbreviations characteristic of the medical idioms (Fig 5A). Filtering out labels not utilising these queries, we tracked the proportion of medical idioms usage across different user-label seniority levels (Fig 5B). Here, the seniority goes up to 15 because, after filtering out irrelevant labels, there are not many user-label pairs with high seniority left in our dataset. Interestingly, the usage of medical idioms appears substantial at the first user-label seniority level, accounting for over 80% of queries. However, this proportion exhibited a decline, stabilising around 35% after the fifth user-label seniority level (zones B and C in Fig 5).

**Fig 6:**
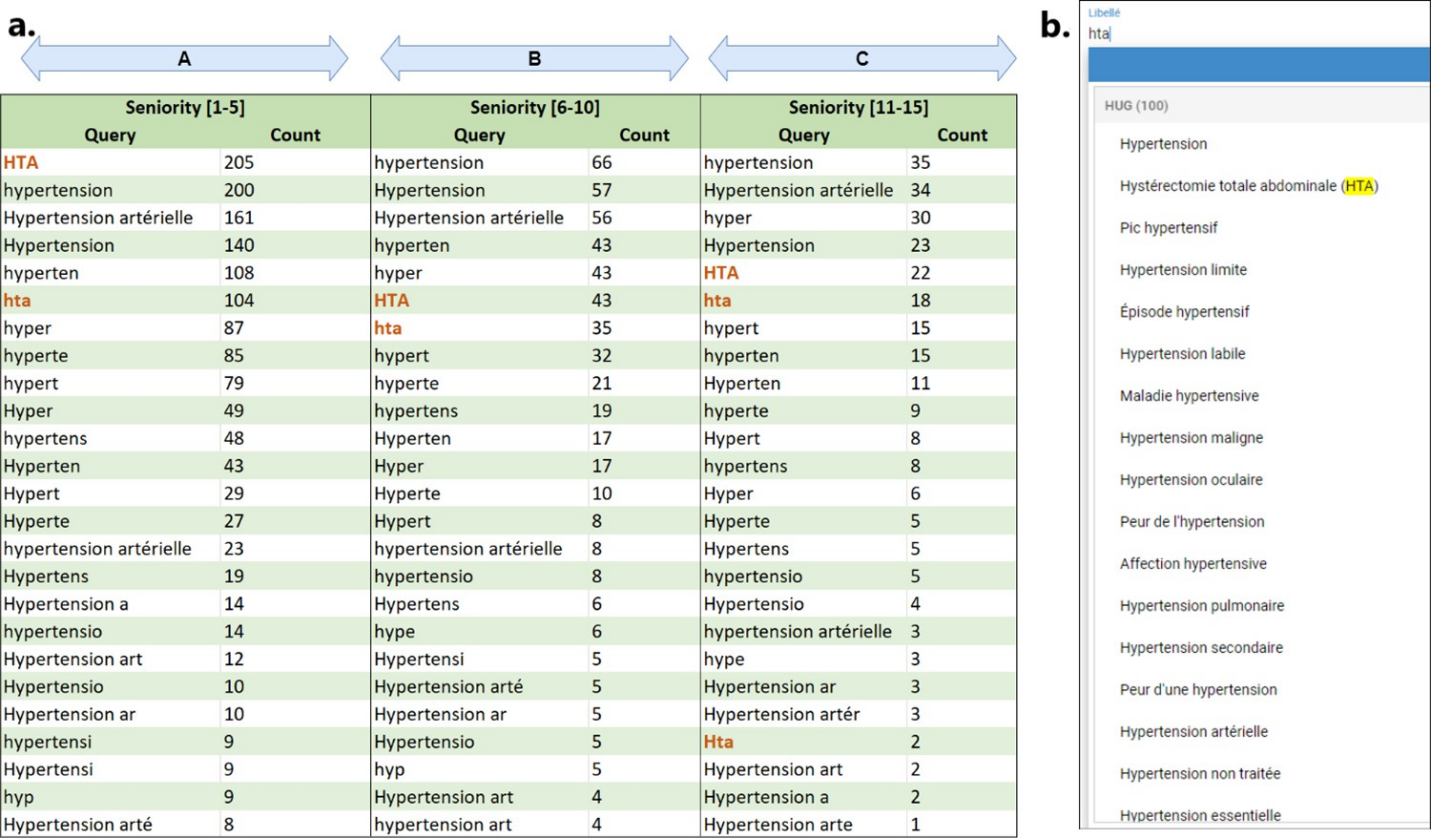
Medical idiom: The case of the query ‘hta’. a. Statistics of queries used to select the label ‘Hypertension Artérielle’ with respect to the seniority (queries in red are part of the medical idiom). b. Results of the query ‘hta’, with the expected label ‘Hypertension Artérielle’ ranked 15th.

While medical idioms facilitate concise communication among healthcare professionals, their incorporation into phrase prediction tools can lead to numerous and sometimes less relevant candidate labels. Fig 6 highlights the issue with the most frequently used query from the medical jargon: “*hta*”. This query predominantly occurs within users’ 1st to 5th selections (the A zone in Fig 6A) associated with the label “*Hypertension artérielle*”. Despite the expression’s high popularity in the medical sector, the expected label is ranked 15th with this query (Fig 6B). Subsequently, users tend to switch to the query "*hypertension*" for subsequent selections (in the B and C zones), where the label is available at a more favourable rank of two.

## Discussion

Our study examines quantitatively and at large scale users’ linguistic adaptations while utilising a medical phrase prediction tool, shedding light on their linguistic strategies to overcome technological barriers. Notably, our findings underscore crucial aspects regarding the pressures that drive the user adaptation to refine the interactions. Our research predominantly delineates two key outcomes.

The first major finding of the study is the confirmation of Zipf’s principle of least effort on a large-scale observation study. As Fig 3 illustrates a correlation between user seniority level and the proximity of queries to the Pareto front, we highlight the adaptive nature of user behaviour, wherein users gravitate towards utilising queries closer to the optimal set defined by the properties of conciseness and distinctiveness. This trend indicates a deliberate effort by users to refine their interactions for a smoother communication. Furthermore, as seniority level increases, the standard deviation decreases, resulting in a more consistent average distance between queries and the optimal set across users and labels. While the interactions may not converge precisely towards the Pareto front, there is a noticeable convergence point in proximity to it. Through familiarity with the tool’s suggested phrases, users adapt their queries based on the principles of efficiency (conciseness) and accuracy (distinctiveness). These properties encapsulate the effort needed to retrieve labels, supporting the hypothesis that humans adapt their linguistic communication with technology in line with Zipf’s least effort principle.

Although it was expected that users would prefer shorter queries, our second significant finding indicates a preference for more distinctive queries driven by accuracy. That is, Fig 4 suggests that the user adaptation process primarily hinges on the ranking of the selected phrases. This observed behaviour might be attributed to users’ willingness to prioritise the reduction of visual attention and cognitive effort while scanning label lists, rather than solely aiming to minimise keyboard usage time [45]. The act of typing might hold less significance compared to the effort involved in navigating through the candidate labels list. Moreover, there seems to be a higher likelihood of user satisfaction when the expected label ranks within the top five positions, as indicated by the mean label rank plot in the latest seniority levels. This finding is consistent with prior research that employed eye-tracking techniques to analyse user behaviour in search results, where fixation times were observed to decrease in a top-down fashion [56–58]. The primacy of communication accuracy in user behaviour is supported by our experiments involving the medical idioms. Some of them diminish in usage as users become more familiar with associated labels (Fig 5). While medical idioms are typically effective and clear within the medical domain, integrating such idiomatic expressions into the query construction is not necessarily optimal when using the phrase prediction tool. This assumption is supported by the qualitative experiment involving the case of “*hta*”, depicted in Fig 6. The aforementioned query is commonly employed in the medical community to denote elevated blood pressure levels (*Hypertension artérielle* in French). A significant observation is that the labels proposed by the query “*hta*” relate to a specific manifestation or condition of hypertension, with the exception of one label, *Hystérectomie Totale Abdominale* (meaning *Total Abdominal Hysterectomy*), which is primarily used by gynaecologists. Hence, it appears that users modify this behaviour by utilising the “*hypertension*” query instead to be able to select the “*Hypertension artérielle*” label at the second rank. As we shift between contexts, from real-world medical conversations among individuals to using a phrase prediction tool in our experiment, there is a notable change in the predictability and informational value of the term “*hta*”. In this transition, the isolated use of “*hta*” experiences a clear decrease in its informational value. This is due to the tool’s consideration of numerous relevant phrases associated with elevated blood pressure. This semantic shift in the understanding of concepts between healthcare professionals and the technology seems to cause the users to adapt their linguistic interactions accordingly. Humans sharpen their current language to use optimal queries that are drawn from either the French language or the medical jargon, altering their vocabulary based on the system’s feedback and rewards.

This work provides insights into how humans optimise the use of language in the context of user adaptation. Building upon a previous investigation^17^ that established a relationship between the principle of least effort and the optimisation of form-meaning mappings that occur under the joint pressures of communication efficiency and accuracy, our study not only confirms this relationship but extends it to a real-world scenario through a large-scale observation study in a clinical setting. Specifically, our examination involved 1,763 healthcare professionals using French language and medical jargon with 10,774 different labels, contrasting with the prior study’s artificial lexicon involving only three words and 124 participants in an experimental setup. Moreover, a crucial distinction arises in our results: humans learn to employ more accurate queries with a minimal impact on communication ease (typically typing one or two extra characters on average). The formation of an optimal lexicon implies that the principle of least effort, along with cultural, cognitive, and social factors, can be a determining element in our linguistic behaviour. The outcomes support the notion that Zipf’s Law can be theoretically explained by the principle of least effort. This is evidenced by the power-law behaviour of the log-log plots of the rank-frequency distributions of words and queries, as detailed in Section 1 of S1 File. Beyond the theoretical and linguistic perspectives, our results argue for the rehabilitation of longer but better-understood sentences in human-machine interfaces, including conversational AI models. Prompt engineering techniques are a perfect consequence of this: users write one or more extra sentences, even if it means giving long, explanatory examples of the task the AI chatbot needs to perform, so that its answer meets the user’s expectations. This paradigm shift not only improves user experiences, but also suggests a re-evaluation of information retrieval methodologies. The adoption of accuracy as a core principle allows for the restructuring of technological interfaces and enhancing how information is efficiently accessed across various fields.

It is important to acknowledge several biases within the methodology rooted in the clinical context. Firstly, the presence of a technology obligation bias exists: hospital employees are mandated to utilise the tool during their professional activities, forcing user acceptance. Additionally, a speed bias emerges as clinicians aim to limit time spent using the tool. Lastly, the correctness bias dictates the necessity for selected phrases to be highly precise and to reflect reality as reliably as possible, for obvious medical reasons. As a result, the broader applicability of this phenomenon to various communication contexts necessitates further investigation due to these inherent biases present in the clinical setting. The study focusing on a specific phrase prediction system, replicating this study in diverse human-machine interaction scenarios using free-text language, beyond the clinical domain and phrase prediction tools, would be beneficial to explore the generalizability of these findings.

Although our study focuses on the French language, the study setting is applicable in any language where efficiency can be quantified by the number of keystrokes inputted. We specifically use textual length as our measure of conciseness, since each character in French corresponds to one keystroke. For languages without this one-to-one correspondence, such as Chinese, the number of keystrokes need to be tracked to measure efficiency.

In traditional communication, speakers and hearers are distinct individuals with differing goals: speakers prioritize conciseness, while hearers focus on clarity [59]. Human-machine interactions, however, differ fundamentally depending on the interaction framework. User-centered interfaces foster asymmetrical communication, while dialog-oriented technologies create symmetrical interactions by making the machine act as a hearer [60]. Our study examines asymmetrical interactions, granting users a dual role that allows them to control their desired level of accuracy and adjust conciseness as needed. This shift fundamentally differs from traditional communication dynamics and might influence the observed preference for distinctiveness, as users can prioritize accuracy and tailor their queries without the uncertainty inherent in interpreting another individual’s perspective.

In conclusion, this study investigated whether humans, here healthcare professionals, adapt their language behaviour according to Zipf’s least effort principle when using a technology demanding natural language as input, here a phrase prediction system to select medical labels. For this purpose, we identified the optimal queries with respect to the properties of conciseness and distinctiveness. They correspond to the two competing pressures: communication efficiency and accuracy. The trade-off between these forces is at the root of the optimisation of the users’ language through the principle of least effort. Experienced users have improved their lexicon even if it means sacrificing some efficiency to improve the accuracy of communication, even if it means reducing the use of well-known, efficient medical terms (e.g., *hta*). The results of this study have two major implications. On one hand, the results of the study support Zipf’s least effort principle stating that humans try to communicate with the smallest amount of effort. On the other hand, they have learned to use an optimised lexicon for smoother interactions, prioritising communication accuracy as the key factor behind this optimisation.

### Code availability

The full code used to perform the analysis is available at the following link: https://github.com/JamilProg/ZipfLangAdapt.

## Supporting information

S1 File Supplementary information. Additional information regarding data characteristics, data analysis, statistical analysis, and detailed mathematical formulation of the problem.
(DOCX)
