# Correction: Human-machine interactions with clinical phrase prediction system, aligning with Zipf’s least effort principle?

**DOI:** 10.1371/journal.pone.0319185

**Published:** 2025-02-06

**Authors:** The PLOS One Staff

## Notice of republication

This article was republished on January 29, 2025, to remove irrelevant in-text links that were introduced during the typesetting process. The publisher apologizes for the errors. Please download the article again to view the correct version. The originally published, uncorrected article and the republished, corrected article are provided here for reference.

## Supporting information

S1 File Originally published, uncorrected article. (PDF)

S2 File Republished, corrected article. (PDF)
