# Supplementary material for: Human-machine interactions with clinical phrase prediction system, aligning with Zipf’s least effort principle?
Source: PLoS One. 2024 Dec 31;19(12):e0316177. doi: 10.1371/journal.pone.0316177 (PMC11687647; doi:10.1371/journal.pone.0316177)
Supplement: S1 File — Additional information regarding data characteristics, data analysis, statistical analysis, and detailed mathematical formulation of the problem. (DOCX) [file pone.0316177.s001.docx]

**Supplementary Information**

**Section 1. Rank-frequency distributions of the dataset**

Zipf's Least Effort Principle has often been regarded as a theoretical explanation of Zipf’s Law, stating that there is an inverse relation between frequency and its corresponding rank. Empirical evidence showed that Zipf's Law occurs in various languages [1], and even in non-textual contexts related to the individual or collective human behavior such as population distribution among cities [2] or the distribution of musical notes [3]. Hence, we assess whether our data resulting from human activity follows Zipf’s distribution. Such data includes words, queries, and the selected labels. For each case, we provide the rank-frequency plot in a log-log scale (Figures 1-3), and two tables showing the most and least frequent items (Tables 1-6). The Zipfian distribution being a discrete power law, it is possible to check whether a dataset does not follow Zipf’s distribution by plotting in a log-log scale. It is expected to be a linear graph as it is the characteristic signature of a power law.

**Words**


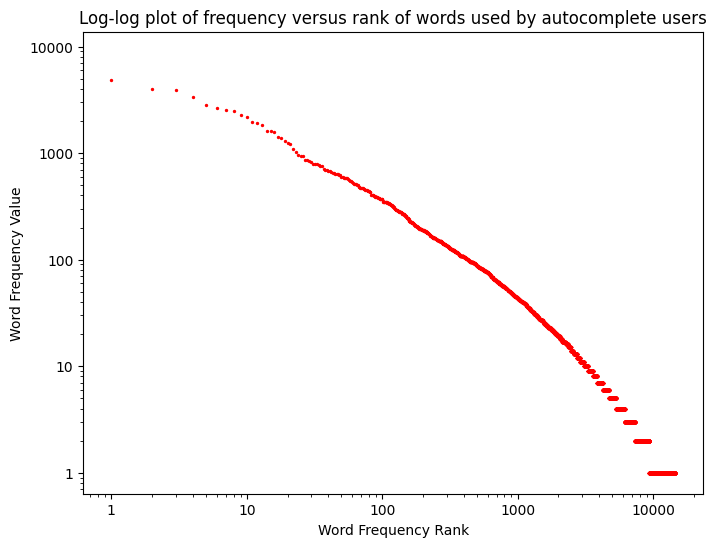


**Fig. 1. Rank-frequency distribution of words (Log-log scale)**

**Table 1. Most frequent words.** The table describes the rank of its frequency, the word and its frequency value.

| **Rank** | **Word** | **Frequency** |  | **Rank** | **Word** | **Frequency** |
| --- | --- | --- | --- | --- | --- | --- |
| 1 | de | 8744 |  | 26 | décompensation | 940 |
| 2 | douleur | 4842 |  | 27 | artérielle | 932 |
| 3 | trouble | 4010 |  | 28 | contusion | 860 |
| 4 | insuffisance | 3886 |  | 29 | prothèse | 854 |
| 5 | fracture | 3339 |  | 30 | crise | 839 |
| 6 | infection | 2832 |  | 31 | d | 832 |
| 7 | du | 2644 |  | 32 | urinaire | 790 |
| 8 | syndrome | 2515 |  | 33 | cardiaque | 788 |
| 9 | anémie | 2497 |  | 34 | covid | 787 |
| 10 | hypertension | 2252 |  | 35 | chronique | 768 |
| 11 | la | 2163 |  | 36 | traumatisme | 756 |
| 12 | etat | 1978 |  | 37 | accident | 754 |
| 13 | chute | 1915 |  | 38 | trauma | 702 |
| 14 | troubles | 1847 |  | 39 | entorse | 695 |
| 15 | hernie | 1623 |  | 40 | obésité | 688 |
| 16 | douleurs | 1611 |  | 41 | sars | 683 |
| 17 | diabète | 1581 |  | 42 | baisse | 681 |
| 18 | rénale | 1412 |  | 43 | en | 659 |
| 19 | à | 1373 |  | 44 | des | 644 |
| 20 | déficit | 1289 |  | 45 | état | 644 |
| 21 | plaie | 1238 |  | 46 | asthme | 639 |
| 22 | maladie | 1222 |  | 47 | hta | 639 |
| 23 | pneumonie | 1100 |  | 48 | fibrillation | 631 |
| 24 | carcinome | 1029 |  | 49 | bilan | 629 |
| 25 | malnutrition | 954 |  | 50 | social | 629 |

**Table 2. Least frequent words.** The table describes the rank of its frequency, the word and its frequency value.

| **Rank** | **Word** | **Frequency** |  | **Rank** | **Word** | **Frequency** |
| --- | --- | --- | --- | --- | --- | --- |
| 14529 | laparot | 1 |  | 14504 | hémib | 1 |
| 14528 | termina | 1 |  | 14503 | pancréatect | 1 |
| 14527 | colosco | 1 |  | 14502 | ovariectomi | 1 |
| 14526 | colono | 1 |  | 14501 | opéra | 1 |
| 14525 | apla | 1 |  | 14500 | nécrosec | 1 |
| 14524 | algoneurodystrophie | 1 |  | 14499 | dive | 1 |
| 14523 | défo | 1 |  | 14498 | hémorroïdec | 1 |
| 14522 | défor | 1 |  | 14497 | hépatect | 1 |
| 14521 | subfebril | 1 |  | 14496 | subocclusion | 1 |
| 14520 | fibromya | 1 |  | 14495 | nyst | 1 |
| 14519 | névralgi | 1 |  | 14494 | tubo-ovarien | 1 |
| 14518 | tumor | 1 |  | 14493 | psychotiqu | 1 |
| 14517 | névralg | 1 |  | 14492 | aphteuse | 1 |
| 14516 | myélopath | 1 |  | 14491 | inferieure | 1 |
| 14515 | sche | 1 |  | 14490 | d'éventr | 1 |
| 14514 | scheuer | 1 |  | .. | .. | .. |
| 14513 | inégal | 1 |  | 9043 | stiff | 2 |
| 14512 | iné | 1 |  | 9042 | transplantatio | 2 |
| 14511 | conis | 1 |  | 9041 | aspira | 2 |
| 14510 | gonflemen | 1 |  | 9040 | cardiom | 2 |
| 14509 | d'évent | 1 |  | 9039 | béta | 2 |
| 14508 | fil | 1 |  | 9038 | leucocyto | 2 |
| 14507 | dyspho | 1 |  | 9037 | toxicité | 2 |
| 14506 | duodéno-pancréatectomie | 1 |  | 9036 | paragangliome | 2 |
| 14505 | doli | 1 |  | 9035 | acouphe | 2 |

## **Queries**


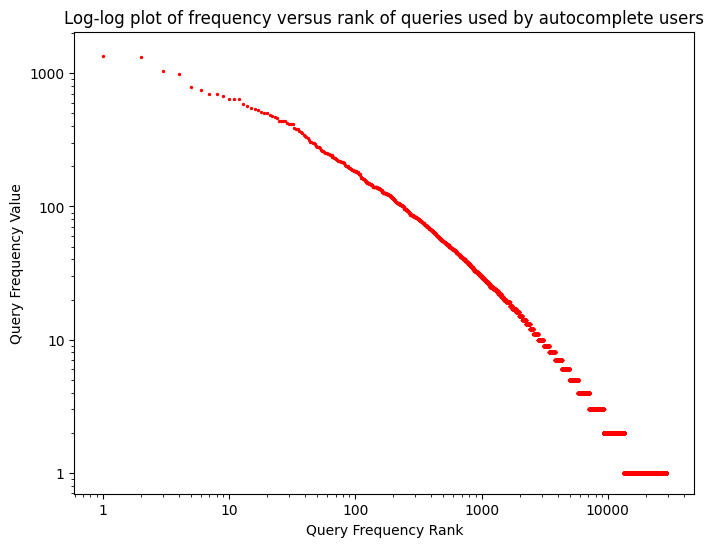


**Fig. 2. Rank-frequency distribution of queries (Log-log scale)**

**Table 3. Most frequent queries.** The table describes the rank, the query and its frequency value.

| **Rank** | **Query** | **Frequency** |  | **Rank** | **Query** | **Frequency** |
| --- | --- | --- | --- | --- | --- | --- |
| 1 | chute | 1428 |  | 26 | hyperten | 441 |
| 2 | douleur | 1352 |  | 27 | contusion | 439 |
| 3 | anémie | 1336 |  | 28 | trauma | 438 |
| 4 | infection | 1039 |  | 29 | syndrome | 438 |
| 5 | hypertension | 986 |  | 30 | entorse | 420 |
| 6 | fracture | 783 |  | 31 | traumatisme | 419 |
| 7 | covid | 750 |  | 32 | hyponatrémie | 417 |
| 8 | insuffisance | 705 |  | 33 | insuffisance rénale | 415 |
| 9 | pneumonie | 698 |  | 34 | malnutrition | 389 |
| 10 | plaie | 675 |  | 35 | ivrs | 385 |
| 11 | douleurs | 641 |  | 36 | infection urinaire | 379 |
| 12 | hta | 639 |  | 37 | vertiges | 370 |
| 13 | diabète | 638 |  | 38 | angine | 364 |
| 14 | hypertension artérielle | 584 |  | 39 | mycose | 358 |
| 15 | etat | 568 |  | 40 | lombalgie | 342 |
| 16 | dyspnée | 546 |  | 41 | hernie | 340 |
| 17 | obésité | 543 |  | 42 | insuffi | 330 |
| 18 | trouble | 532 |  | 43 | hypert | 329 |
| 19 | hyper | 515 |  | 44 | céphalées | 315 |
| 20 | déficit | 502 |  | 45 | gastrite | 306 |
| 21 | asthme | 500 |  | 46 | douleur thoracique | 303 |
| 22 | social | 484 |  | 47 | malnutr | 301 |
| 23 | sars | 475 |  | 48 | bilan | 298 |
| 24 | malaise | 471 |  | 49 | bpco | 297 |
| 25 | avc | 460 |  | 50 | tabagisme | 285 |

**Table 4. Least frequent queries.** The table describes the rank, the query and its frequency value.

| **Rank** | **Query** | **Frequency** |  | **Rank** | **Query** | **Frequency** |
| --- | --- | --- | --- | --- | --- | --- |
| 29005 | appendicectomie la | 1 |  | 28980 | hernie ombilicale incarc | 1 |
| 29004 | defibri | 1 |  | 28979 | oedème ai | 1 |
| 29003 | décompensation respir | 1 |  | 28978 | syndrome ext | 1 |
| 29002 | dav | 1 |  | 28977 | deliriu | 1 |
| 29001 | perturbation test hepat | 1 |  | 28976 | injection de bo | 1 |
| 29000 | apport en éner | 1 |  | 28975 | microt | 1 |
| 28999 | malnutrition protéino-énergétiqu | 1 |  | 28974 | migraine av | 1 |
| 28998 | malnutrition protéino-énergétique lége | 1 |  | 28973 | todd | 1 |
| 28997 | eveinage | 1 |  | 28972 | paralysie faciale péri | 1 |
| 28996 | risque d'apport énergétique sous-optimal | 1 |  | 28971 | phé | 1 |
| 28995 | prothese mam | 1 |  | 28970 | lésion m | 1 |
| 28994 | plaie trau | 1 |  | 28969 | céphalées chroni | 1 |
| 28993 | continui | 1 |  | 28968 | clus | 1 |
| 28992 | dysfonction cardiaque | 1 |  | 28967 | hun | 1 |
| 28991 | abcés cervical | 1 |  | 28966 | myoméctomie | 1 |
| 28990 | abcès péri amygdalien | 1 |  | .. | .. | .. |
| 28989 | adénocarcinome oes | 1 |  | 13473 | doigt de | 2 |
| 28988 | poids insuffi | 1 |  | 13472 | accident vasculaire ischémique aig | 2 |
| 28987 | adénocarcinome tubulaire | 1 |  | 13471 | colite à c. | 2 |
| 28986 | état confusionnel aigü | 1 |  | 13470 | accident vasculaire cérébral ischémique aig | 2 |
| 28985 | ddimères | 1 |  | 13469 | obésité de classe 2 | 2 |
| 28984 | fracture coraco | 1 |  | 13468 | accident vasculaire trans | 2 |
| 28983 | fracture condyl | 1 |  | 13467 | erythème toxique | 2 |
| 28982 | volet | 1 |  | 13466 | apnée du sommei | 2 |
| 28981 | syncope v | 1 |  | 13465 | cross | 2 |

**Selected labels**


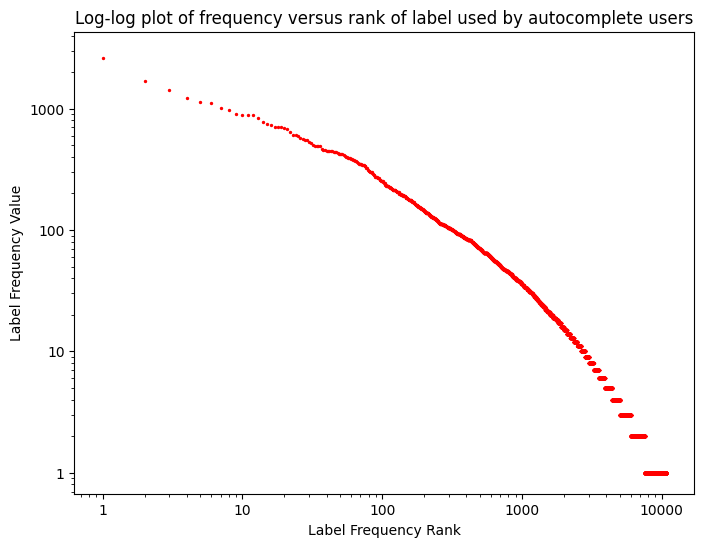


**Fig. 3. Rank-frequency distribution of selected labels (Log-log scale)**

**Table 5. Most frequently selected labels.** The table describes the rank, the label and its frequency value.

| **Rank** | **Selected label** | **Frequency** |  | **Rank** | **Selected label** | **Frequency** |
| --- | --- | --- | --- | --- | --- | --- |
| 1 | hypertension artérielle | 2886 |  | 26 | malnutrition protéino-énergétique modérée | 594 |
| 2 | infection à sars-cov-2 (covid19) | 2611 |  | 27 | fracture | 571 |
| 3 | déficit de force avec perte de fonctionnalité | 1693 |  | 28 | traumatisme crânien simple | 566 |
| 4 | insuffisance rénale aiguë | 1418 |  | 29 | infection urinaire | 556 |
| 5 | hypertension artérielle traitée | 1232 |  | 30 | fibrillation auriculaire | 546 |
| 6 | anémie normochrome normocytaire | 1141 |  | 31 | baisse de l'état général | 526 |
| 7 | médecine préventive | 1122 |  | 32 | dyslipidémie | 516 |
| 8 | douleur thoracique | 1011 |  | 33 | diabète type 2 | 499 |
| 9 | douleur abdominale | 974 |  | 34 | prévention vaccinale sars-cov-2 (covid19) | 496 |
| 10 | bilan social | 898 |  | 35 | chute chez la personne âgée | 494 |
| 11 | troubles de la déglutition | 891 |  | 36 | perturbation des tests hépatiques | 494 |
| 12 | douleurs abdominales | 884 |  | 37 | reflux gastro-oesophagien | 491 |
| 13 | hypercholestérolémie | 879 |  | 38 | infection des voies respiratoires supérieures | 468 |
| 14 | insuffisance rénale chronique | 840 |  | 39 | vertiges | 457 |
| 15 | troubles cognitifs | 776 |  | 40 | état fébrile d'origine indéterminée | 453 |
| 16 | état confusionnel aigu | 745 |  | 41 | traumatisme crânien | 451 |
| 17 | accouchement normal d'un nouveau-né vivant par voie basse | 737 |  | 42 | pneumonie acquise en communauté | 449 |
| 18 | appendicectomie | 709 |  | 43 | insuffisance respiratoire hypoxémique | 445 |
| 19 | décompensation cardiaque | 706 |  | 44 | asthme | 445 |
| 20 | malnutrition protéino-énergétique grave | 701 |  | 45 | infection urinaire basse | 444 |
| 21 | tabagisme actif | 688 |  | 46 | hypovitaminose d | 441 |
| 22 | hyponatrémie | 675 |  | 47 | décompensation cardiaque globale | 438 |
| 23 | hypokaliémie | 643 |  | 48 | céphalées | 435 |
| 24 | état fébrile | 608 |  | 49 | épigastralgie | 431 |
| 25 | dyspnée | 607 |  | 50 | douleurs thoraciques | 426 |

**Table 6. Least frequently selected labels.** The table describes the rank, the label and its frequency value.

| **Rank** | **Selected label** | **Frequency** |  | **Rank** | **Selected label** | **Frequency** |
| --- | --- | --- | --- | --- | --- | --- |
| 10777 | sténose calcifiée de l'aorte | 1 |  | 10752 | réparation d'une déchirure périnéale de degré 3c | 1 |
| 10776 | tumeur bénigne de l'utérus | 1 |  | 10751 | compression du nerf cubital | 1 |
| 10775 | défaillance cardiaque avec décompensation | 1 |  | 10750 | bronchopneumopathie chronique obstructive clinique | 1 |
| 10774 | histiocytose x | 1 |  | 10749 | corne cutanée | 1 |
| 10773 | recherche du hpv | 1 |  | 10748 | thalassémie hétérozygote | 1 |
| 10772 | hidrosadénite | 1 |  | 10747 | tumeur maligne du pancréas | 1 |
| 10771 | fracture du talon | 1 |  | 10746 | syndrome de dépendance aux sédatifs et aux tranquillisants non barbituriques mais aux effets comparables | 1 |
| 10770 | echographie | 1 |  | 10745 | syndrome de dépendance aux sédatifs | 1 |
| 10769 | diverticulite avec complication | 1 |  | 10744 | insuffisance cardiaque gauche et probablement droite | 1 |
| 10768 | dermatite chronique | 1 |  | 10743 | épuisement dû à la déshydratation par la chaleur | 1 |
| 10767 | cure de doigt à ressort | 1 |  | 10742 | syndrome extra-pyramidal secondaire à la prise d'un médicament | 1 |
| 10766 | fracture osseuse | 1 |  | 10741 | larmoiement | 1 |
| 10765 | consommation nocive de cannabinoïdes | 1 |  | 10740 | embolie pulmonaire lobaire moyenne | 1 |
| 10764 | rhinite saisonnière | 1 |  | 10739 | décompensation cardiaque gauche sur tachyarythmie | 1 |
| 10763 | bilan respiratoire | 1 |  | 10738 | cystite interstitielle chronique | 1 |
| 10762 | arthroplastie trapézo-métacarpienne | 1 |  | .. | .. | .. |
| 10761 | ancienne toxicomanie sevrée | 1 |  | 7563 | ablation de fils | 2 |
| 10760 | affection du ménisque | 1 |  | 7562 | tétralogie de fallot congénitale | 2 |
| 10759 | cellulite orbitaire | 1 |  | 7561 | tronc artériel commun d'origine congénitale | 2 |
| 10758 | abus sexuel | 1 |  | 7560 | sarcome indifférencié | 2 |
| 10757 | fracture pertrochantérienne du fémur traitée par dhs | 1 |  | 7559 | caries multiples | 2 |
| 10756 | abcès de la cuisse | 1 |  | 7558 | scoliose acquise | 2 |
| 10755 | trait drépanocytaire | 1 |  | 7557 | syndrome d'hypoventilation sur obésité | 2 |
| 10754 | porteur de valve prothétique | 1 |  | 7556 | arthrite à microcristaux | 2 |
| 10753 | sténose trachéale | 1 |  | 7555 | petite taille et petit poids pour l'âge gestationnel | 2 |

#

**Section 2. Additional examples of Pareto Fronts**

In addition to the Pareto front shown for the “Carcinome du pancréas” label, we show some additional cases (Figures 4-8) that use the medical idiom (pnp, hta, bpco, avp). Some of them do not have a Pareto curve, as there is one single optimal point (Figures 6-8).


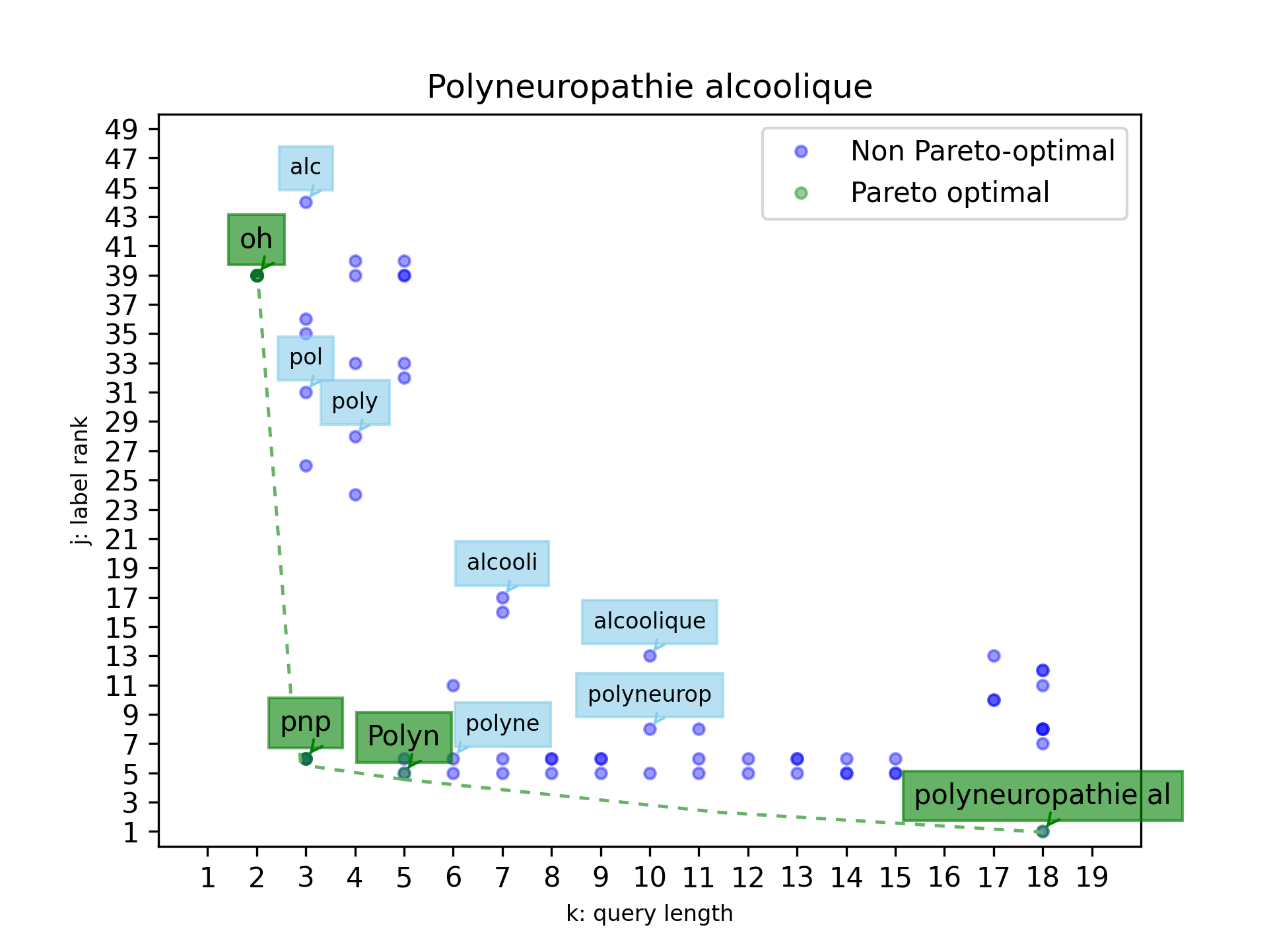


**Fig. 4. Pareto front of the label Polyneuropathie alcoolique**


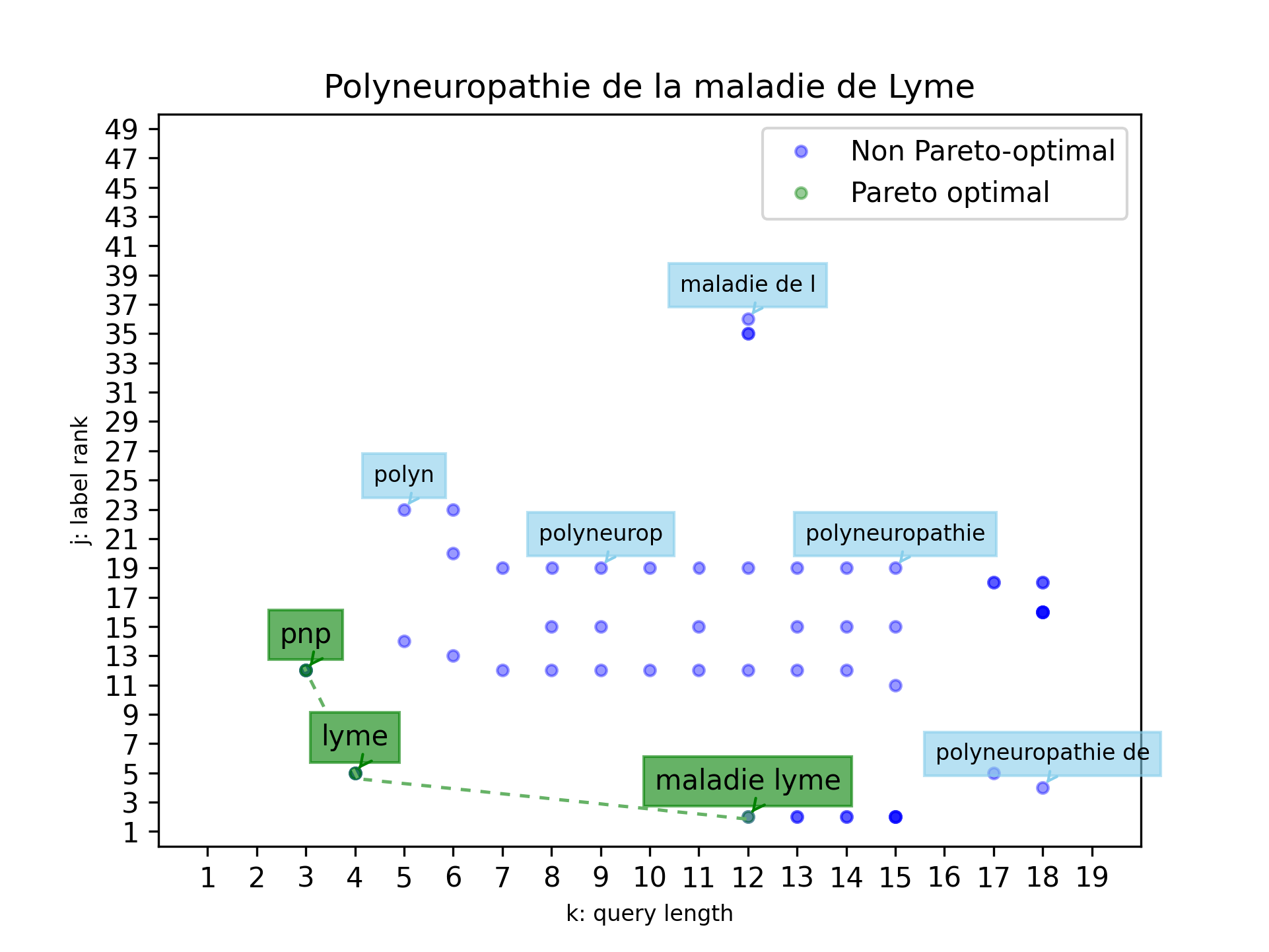


**Fig. 5. Pareto front of the label Polyneuropathie de la maladie de Lyme**


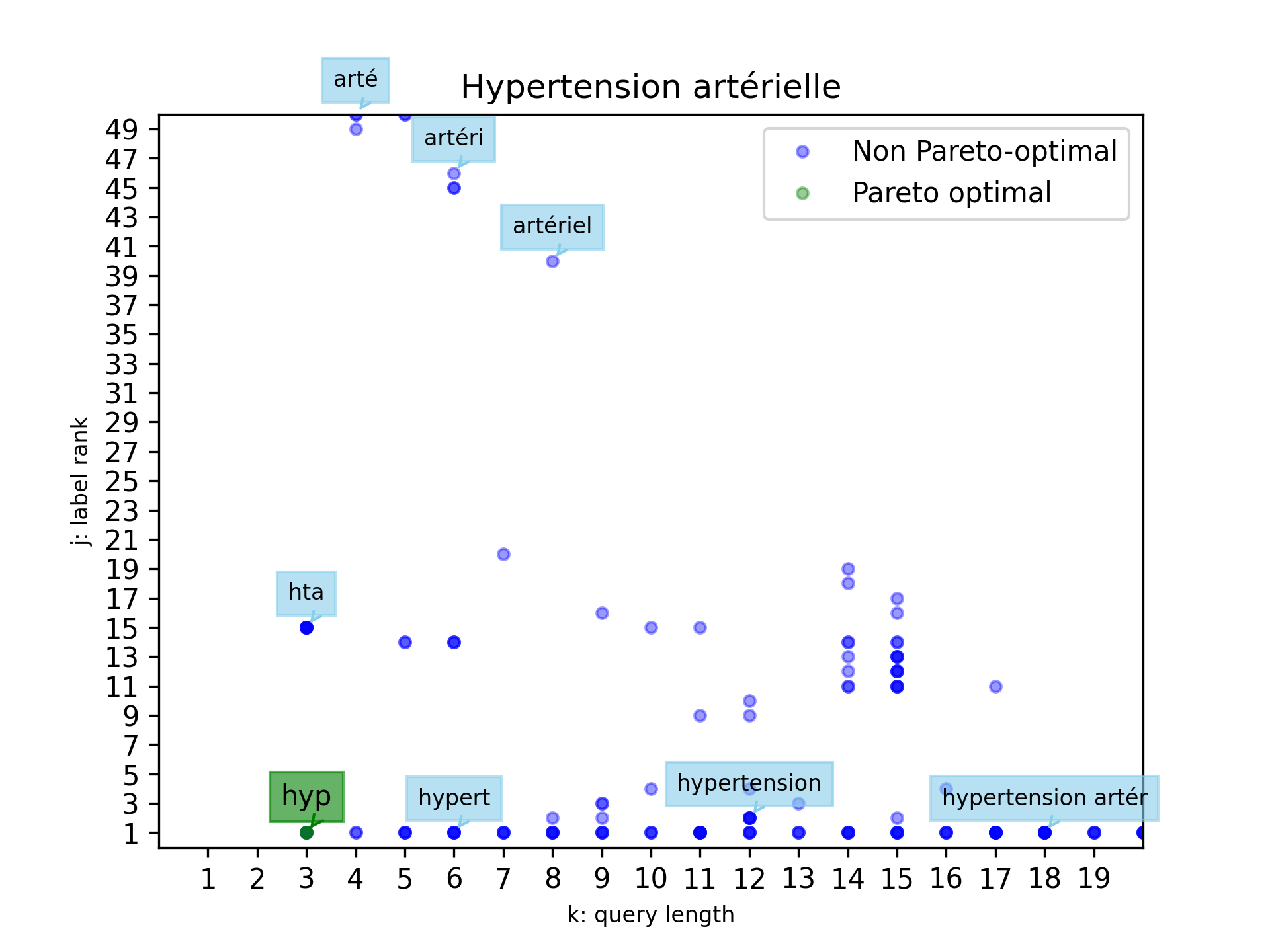


**Fig. 6. Pareto front of the label Hypertension artérielle**


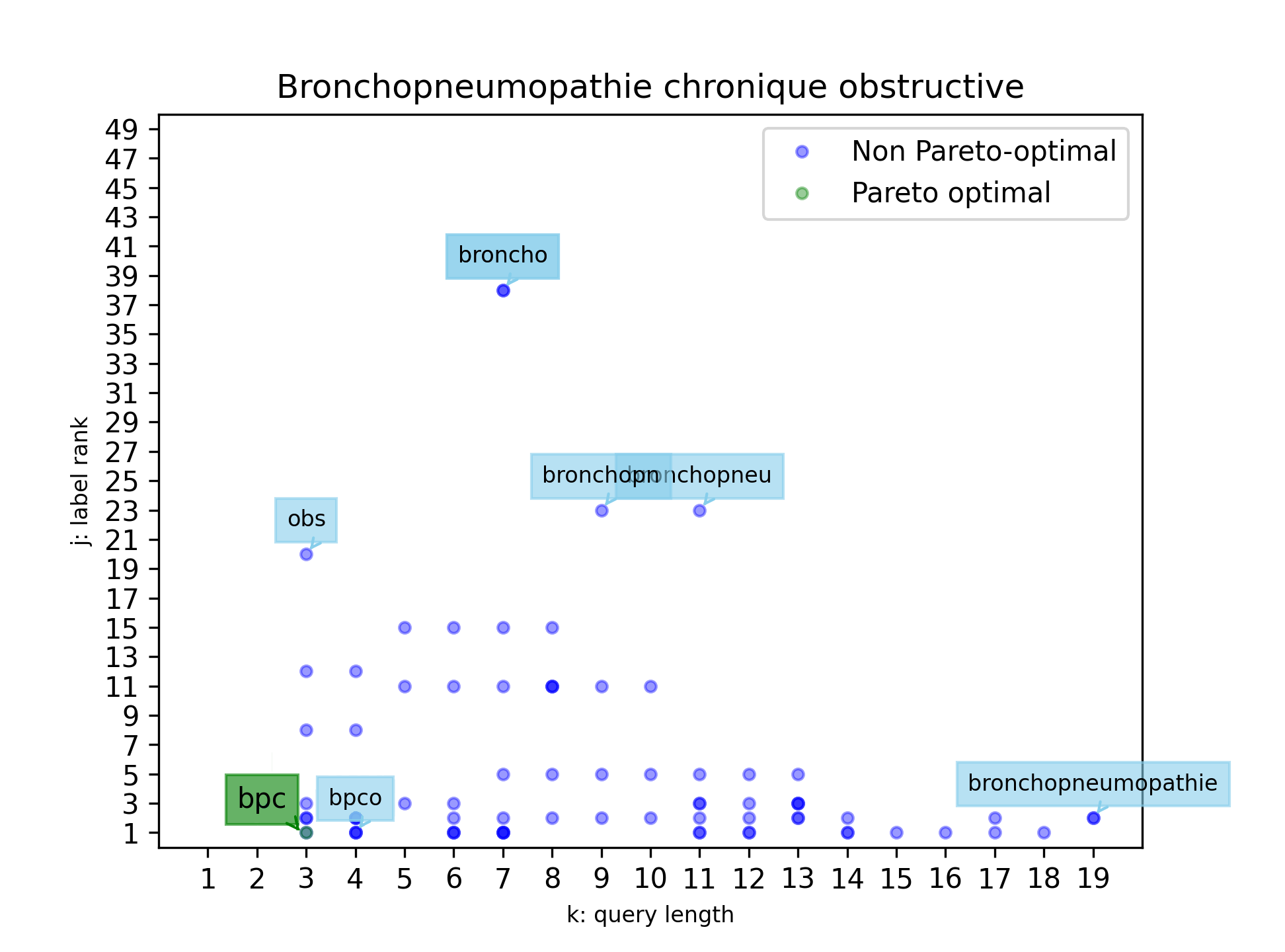


**Fig. 7. Pareto front of the label Bronchopneumopathie chronique obstructive**


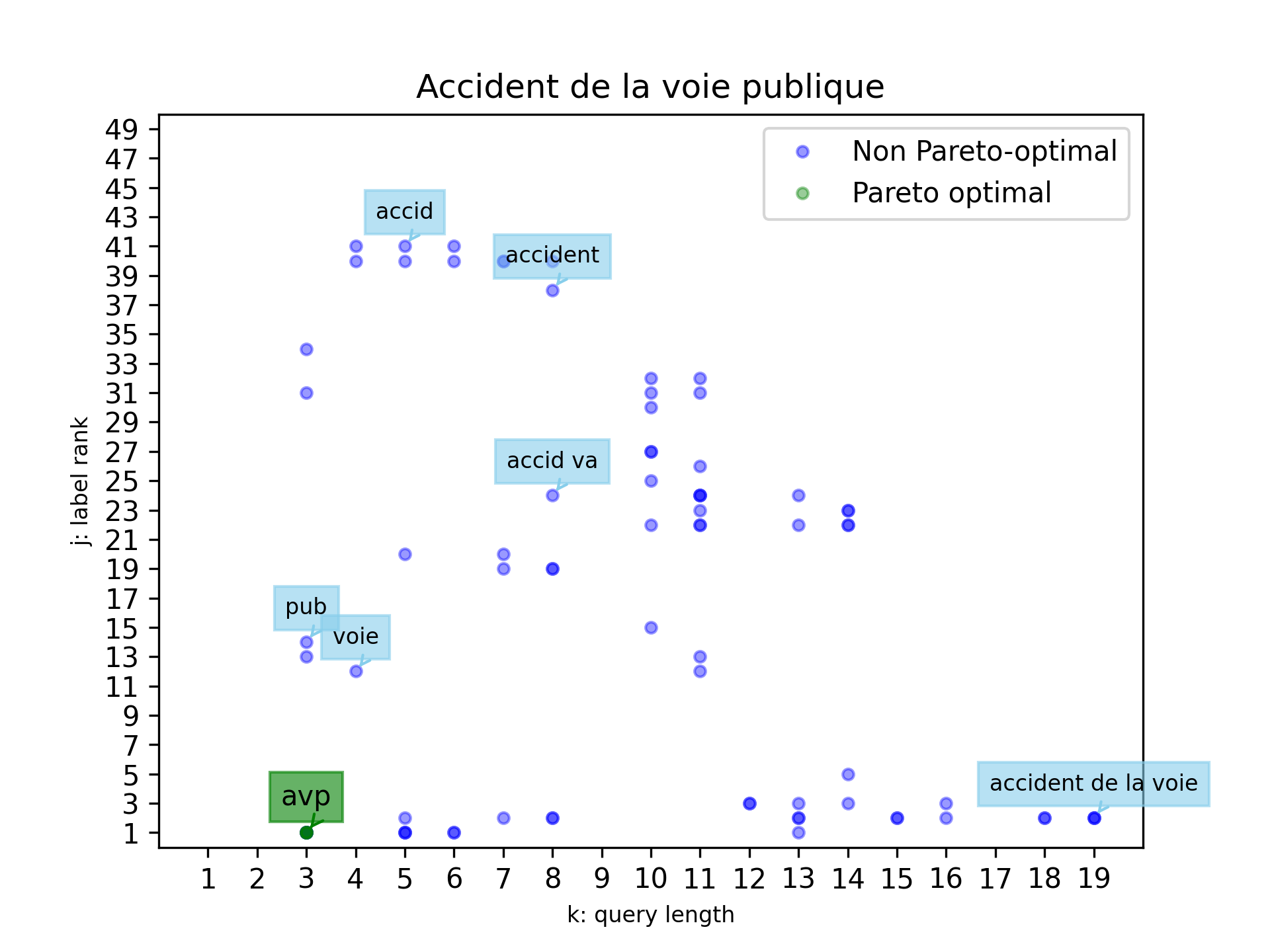


**Fig. 8. Pareto front of the label Accident de la voie publique**

# **Section 3. Statistical analysis of the sample size**

To perform MOO analysis without label variation bias, we excluded user-label pairs with labels that did not attain the highest user-label seniority. The initial population size comprised 100,087 user-label pairs. The sample size after exclusion varied according to the maximum user-label seniority level: higher values of this parameter resulted in fewer labels and user-label pairs reaching that level. We applied the following formula to estimate the minimum sample size [4]:

$n= \left\lceil\frac{N \times\left( \frac{z_{\alpha/2}^{2}\times p \times\left( 1 - p \right)}{m^{2}} \right)}{\left( \frac{z_{\alpha/2}^{2}\times p \times\left( 1 - p \right)}{m^{2}} \right)+ N - 1} \right\rceil$ (1)

With [
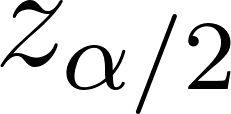
](https://www.codecogs.com/eqnedit.php?latex=z_%7B%5Calpha%2F2%7D#0), [
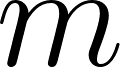
](https://www.codecogs.com/eqnedit.php?latex=m#0), [
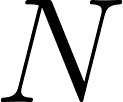
](https://www.codecogs.com/eqnedit.php?latex=N#0) and [
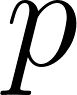
](https://www.codecogs.com/eqnedit.php?latex=p#0) being, respectively, the critical value of the normal distribution at α/2 (for a confidence level of 95%, α is 0.05 and the critical value is 1.96), the margin of error, the population size and the estimation of population proportion (if unknown, 0.5 gives the largest sample size). This equation yielded a minimum sample size of 1,056 for a 95% confidence level and a 3% margin of error. Table 7 shows that this sample size is sufficient for a maximum user-label seniority of 90.

**Table 7. Number of unique user-label pairs after label variation bias elimination with respect to seniority.**

| **Max Seniority** | **1** | **10** | **20** | **30** | **40** | **50** | **60** | **70** | **80** | **90** | **100** |
| --- | --- | --- | --- | --- | --- | --- | --- | --- | --- | --- | --- |
| Sample size | 100087 | 27961 | 12682 | 7490 | 5109 | 3128 | 2033 | 1715 | 1215 | 1211 | 554 |

# **Section 4. Detailed mathematical formulation of the problem**

The mathematical formulation of the MOO problem is defined as follows:

**Definition 1:** $\boldsymbol{\Phi}$, called **alphabet**, is a set of expressible symbols, with $\boldsymbol{\Phi=}\{a-z\} \cup\{0-9\} \cup\{space\} \cup\{-\}$. Hence, $\left| \boldsymbol{\Phi} \right|$ = 38.

**Definition 2:** $\mathbb{T}$, called **terminology**, is a set containing expressions. Among elements from that set, $t_{1}$, $t_{2}$,...,$t_{\mathbb{|T|}}$, called **labels**, $L$ is the length of the longest label.

$\Phi^{k}$ is defined as a set containing permutations with repetition of elements from $\boldsymbol{\Phi}$ of length k (e.g., *hello* $\in\Phi^{5}$). For a set containing $n$ elements, as there are $n^{k}$ possible permutations with repetition of length $k$, according to **Definition 1**, the size of the set is $\left| \Phi^{k} \right|= {\left| \Phi\right|^{k}= 38^{k}}$. We define $\boldsymbol{\Phi}^{\boldsymbol{*}}$ as a set containing the union of sets $\boldsymbol{\Phi}^{\boldsymbol{k}}$ for $\forall k\in\mathbb{N}^{*}, k\leq L$, i.e., $\boldsymbol{\Phi}^{\boldsymbol{*}}$ is the set of all possible strings up to the maximum length L. Formally:

$\boldsymbol{\Phi}^{\boldsymbol{*}}\boldsymbol{=}\cup_{\boldsymbol{i=1}}^{\boldsymbol{L}} \boldsymbol{\Phi}^{\boldsymbol{i}}$ (1)

According to (1):

$\left| \boldsymbol{\Phi}^{\boldsymbol{*}} \right|\boldsymbol{=}\sum_{\boldsymbol{i=1}}^{\boldsymbol{L}} \left| \boldsymbol{\Phi}^{\boldsymbol{i}} \right|\boldsymbol{=}\sum_{\boldsymbol{i=1}}^{\boldsymbol{L}} \left| \boldsymbol{\Phi} \right|^{\boldsymbol{i}}\boldsymbol{=}\sum_{\boldsymbol{i=1}}^{\boldsymbol{L}} \boldsymbol{3}\boldsymbol{8}^{\boldsymbol{i}}$ (2)

According to (2), and the properties of geometric series:

$\left| \boldsymbol{\Phi}^{\boldsymbol{*}} \right|\boldsymbol{=}\sum_{\boldsymbol{i=1}}^{\boldsymbol{L}} \boldsymbol{3}\boldsymbol{8}^{\boldsymbol{i}}\boldsymbol{=}\left( \sum_{\boldsymbol{i=0}}^{\boldsymbol{L}} \boldsymbol{3}\boldsymbol{8}^{\boldsymbol{i}} \right)\boldsymbol{-1 =}\frac{\boldsymbol{1- 3}\boldsymbol{8}^{\boldsymbol{L+1}}}{\boldsymbol{1-38}}\boldsymbol{-1}$ (3)

We denote $\boldsymbol{A}_{\mathbb{T}}$ as the set containing all possible sequences without repetition of labels from $\mathbb{T}$, since no label should be suggested twice by the prediction system. Additionally, ${\boldsymbol{A}_{\mathbb{T}}}^{\boldsymbol{J}}$ is the set of sequences from $\boldsymbol{A}_{\mathbb{T}}$ of size $\boldsymbol{J, \forall J\in} \mathbb{N}^{*}, J\leq\left| \mathbb{T} \right|$.

The **phrase prediction function** is defined as the following: $F :\boldsymbol{\Phi}^{\boldsymbol{*}}\boldsymbol{\mapsto}{\boldsymbol{A}_{\mathbb{T}}}^{\boldsymbol{J}}$. The $\boldsymbol{j}^{\boldsymbol{th}}$ element outputted by $F(x)$, $x\in\boldsymbol{\Phi}^{\boldsymbol{*}}$, is denoted as $F(x)_{j}$, with $F(x)_{j}\mathbb{\in T}$and $j \in\mathbb{N}^{*}, j\leq J$.

Finally, we denote $\mathbb{P}^{*}$as the solution space of the MOO problem. It contains elements from $\boldsymbol{\Phi}^{\boldsymbol{*}}$, which have already occurred at least once empirically in the phrase prediction database recording activities, hence $\mathbb{P}^{*}\subset\Phi^{*}$. Similarly, $\mathbb{P}^{k}$ is a subset of $\mathbb{P}^{\boldsymbol{*}}$, whose permutations are of length k. Hence, $\forall k\in\mathbb{N}^{*}, \mathbb{P}^{k}\subset\mathbb{P}^{*}$.

The **optimum** is defined as the minimisation of two objective functions: the first one minimises the length of the query while the second one tries to obtain the expected label at the best possible rank. The optimum considers only elements from $\boldsymbol{\Phi}^{\boldsymbol{*}}$ that have already occurred at least once in a real-world context, denoted as $\mathbb{P}^{*}$. The optimum of a label $t \in\mathbb{T}$ is a set of elements from $\mathbb{P}^{*}$ respecting the following Multi-Objective Optimisation composed of two objective functions:

$\mathbb{O}_{\mathbb{E}}\left( t \right)=\left\{ \begin{aligned} \mathrm{argmi}n_{p^{k}\in\mathbb{P}^{k}}\left[ \mathrm{mi}n_{k\in\mathbb{N}^{*}}\left( F\left( p^{k} \right) \right) \right] such that \exists j\in\mathbb{N}^{*},j\leq J,F\left( p^{k} \right)_{j}=t \\ \mathrm{argmi}n_{p\in\mathbb{P}^{*}}\left[ \mathrm{mi}n_{j\in N^{*},j\leq J}\left( F\left( p \right)_{j} \right) \right] such that F\left( p \right)_{j}=t \end{aligned} \right.$ (4)

# **References**

1. Piantadosi ST. Zipf’s word frequency law in natural language: A critical review and future directions. Psychonomic bulletin & review. 2014;21:1112–30.

2. Ioannides YM, Overman HG. Zipf’s law for cities: an empirical examination. Regional science and urban economics. 2003;33(2):127–37.

3. Manaris B, Romero J, Machado P, Krehbiel D, Hirzel T, Pharr W, et al. Zipf’s law, music classification, and aesthetics. Computer Music Journal. 2005;29(1):55–69.

4. Daniel WW, Cross CL. Biostatistics: a foundation for analysis in the health sciences. Wiley; 2018.
